# Quality of Life in Patients with Morphea: A Cross-Sectional Study and a Review of the Current Literature

**DOI:** 10.1155/2020/9186274

**Published:** 2020-03-13

**Authors:** Justyna Szczęch, Dominik Samotij, Kamila Jaworecka, Aleksandra Tobiasz, Adam Reich

**Affiliations:** ^1^Department of Dermatology, University of Rzeszow, Rzeszów, Poland; ^2^Students' Scientific Circle of Experimental Dermatology, Department of Dermatology, Venereology and Allergology, Wroclaw Medical University, Wrocław, Poland

## Abstract

**Objective:**

The aim of the study was to evaluate QoL in patients suffering from morphea. *Material and Methods*. Sixty-five patients with morphea were recruited into this cross-sectional, prospective parallel study. QoL among adult patients was assessed with the Dermatology Life Quality Index (DLQI) and Euro-QoL-5D questionnaire; patients aged <17 years used the Children's Dermatology Life Quality Index (CDLQI). The severity of morphea was assessed using the Localized Scleroderma Cutaneous Assessment Tool. The results of QoL and its association with disease severity were compared between patients with various morphea subtypes.

**Results:**

The mean DLQI scoring was 3.8 ± 4.1 points and the CDLQI was 2.3 ± 3.0. The mean value of Visual Analogue Scale thermometer (EQ VAS) was 66.9 ± 17.5 points. The disease activity of morphea based on mLoSSI correlated significantly with QoL impairment according to the DLQI (*R* = 0.41, *p* = 0.001). No significant correlation was observed between morphea-induced damage and QoL (*p* = 0.99).

**Conclusions:**

Evaluation of QoL in patients with morphea is still challenging due to lack of good assessment tools dedicated specifically for morphea patients. In general, QoL in morphea patients is significantly correlated with the disease activity, but not with disease-induced skin damage.

## 1. Introduction

Morphea (also named as localized scleroderma (LS)) is a rare autoimmune inflammatory disease that essentially affects the skin and/or subcutaneous tissue [1]. It is more common in females, with a female to male ratio of 2-4 : 1 depending on the studied population [2]. The etiology of morphea still remains unknown; however, the genetic factors, trauma, and vascular abnormalities are considered major trigger factors of this disease [3].

Morphea has a wide spectrum of clinical involvement starting from a relatively mild severity with single plaques. In some cases, the disease leads to facial deformation or joint contractions followed by severe movement impairment. Due to an ample variety of clinical forms of morphea, there are few slightly different classifications proposed in the literature. One of the most current classification was developed in 2017 by the European Dermatology Forum (EDF) [4]. The main subtypes of the disease distinguished in the EDF classification are limited type, generalized type, linear type, deep type, and mixed type [4]. However, the EDF S1-guideline authors also considered eosinophilic fasciitis as another subtype within the spectrum of LS.

Studies assessing the impact of morphea on QoL are giving rather conflicting results. Both the active disease, with typical “lilac ring” around the plaques, and the residual hypo-/hyperpigmented lesions might cause substantial discomfort for the patient. Therefore, patients with morphea might present different impacts of the disease on QoL in different stages of the disease. The main purpose of this study was to assess the QoL of the patients with morphea. In this paper, authors have also discussed the results of previously published studies that assessed the QoL of morphea patients. Due to the rarity of this disease, the number of the patients in almost all of the previously conducted studies was relatively small.

## 2. Material and Methods

### 2.1. Participants

This was a cross-sectional, prospective study conducted in two dermatology clinics in Poland. A total of 65 patients with morphea (6 patients aged below 17 years) were included. The mean age of the patients was 50.9 ± 20.5 years (range: 7-82 years), and 87.7% were women. Morphea subtype was defined based on the classification proposed in 2017 by the EDF [4]. Patients with lichen sclerosus took part in this study, as this disease is considered a part of a wide spectrum of morphea clinical picture. General patients' characteristics are summarized in Table 1. All patients agreed to take part in the study, and the project was approved by the local ethics committee.

### 2.2. Assessment of Skin Disease Severity

Several assessment tools were proposed to measure the severity of morphea, although to date, the Localized Scleroderma Cutaneous Assessment Tool (LoSCAT) seems to be the only one with assessed validity and reliability [5]. The LoSCAT allows physicians to measure the LS severity in the active stage and to assess the level of damage developed in the course of the disease. The first part of the LoSCAT, a modified Localized Scleroderma Skin Activity Index (mLoSSI), measures the disease severity in the active, inflammatory stage. Summary scores range from 0 to 162, with higher scores indicating more disease activity [6]. The second part is a Localized Scleroderma Skin Damage Index (LoSDI), which evaluates three domains of damage that might develop in the course of morphea; again, the summary scores range from 0 to 162 points, with higher scores indicating more severe damage [5, 6].

Based on overall LoSCAT scoring, patients were divided into 3 subgroups based on the disease severity: mild, moderate, and severe. The activity corresponded with mLoSSI scores of 0-4, 5-12, and 13 and over; and mild, moderate, and severe damage corresponded with LoSDI scores of 0-10, 11-15, and 16 and over, respectively [7]. This process allowed to compare the QoL specific for different disease severity.

### 2.3. Assessment of QoL

To evaluate the patients' QoL, we used the DLQI, a self-administered questionnaire designed for adult patients with skin diseases [8]. For children and teenagers, the analogical questionnaire—Children Dermatology Life Quality Index (CDLQI)—was chosen [9]. In both of them, the score ranges from 0 to 30 with higher scores indicating lower QoL.

Euro-QoL-5D is a standardized instrument used to evaluate the health outcome. It consists of 5 questions that evaluate patients' status in terms of mobility, self-care, usual activities, pain/discomfort, and anxiety/depression. The second part of EQ-5D contains a Visual Analogue Scale thermometer (EQ VAS) in which a patient subjectively evaluates his/her health from 0 (referred as “the worst health you can imagine”) to 100 points (“the best health you can imagine”) [10].

## 3. Results

### 3.1. Disease Onset, Duration, and Clinical Subtypes

The mean disease duration was 3.9 ± 5.7 years; the mean age at onset of the disease was 46.0 ± 21.5 years. Majority of our patients had plaque-type morphea (*n* = 31, 47.7%). Disseminated plaque-type morphea was diagnosed in 15 (23.1%) of all patients, followed by atrophoderma of Pasini and Pierini (*n* = 7, 10.8%), lichen sclerosus (*n* = 5, 7.7%), linear morphea (*n* = 3, 4.6%), progressive facial hemiatrophy (*n* = 2, 3.1%), deep morphea, and linear morphea *en coup de sabre* (*n* = 1, 1.5%).

### 3.2. Disease Severity

**Figure 1:**
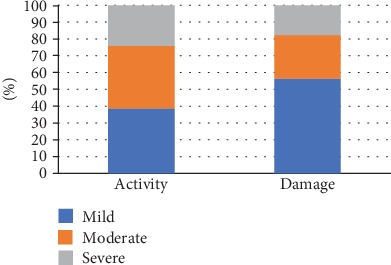
Classification of morphea intensity/severity [10].

The mean mLoSSI was 8.9 ± 9.6 points, and the mean LoSDI was 11.5 ± 10.3 points. Additionally, patients were divided into 3 subgroups based on the severity of both activity and damage. The characteristics of the morphea severity subgroup based on this classification are shown in Figure 1. Majority of patients had mild or moderate intensity of both activity and damage based on mLOSSI and LoSDI scoring (activity: mild—38.7%, moderate—37.1%, and severe—24.2%; damage: mild—56.5%, moderate—25.8%, and severe—17.7%).

### 3.3. Quality of Life Assessment

The mean value of the DLQI among the patients with morphea was 3.7 ± 4.0 points. The mean value of the VAS used in EQ-5D was 66.9 ± 17.5 points. Regarding the first part of EQ-QoL-5D, majority of patients achieved level 1, meaning they had no troubles in domains of mobility, self-care, and usual activities. However, in domains connected with discomfort/pain and anxiety/depression, almost half of the patients with morphea achieved level 2 or level 3 as summarized in Table 2.

### 3.4. QoL and Disease Severity

**Figure 2:**
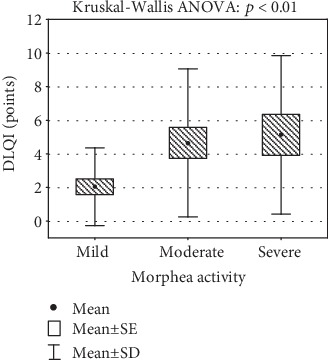
Morphea activity and scoring of the Dermatology Life Quality Index (DLQI).

The disease activity of morphea based on mLoSSI correlated significantly with QoL impairment based on the overall DLQI score (*R* = 0.41, *p* = 0.001). There was no such correlation between damage in the course of morphea and DLQI (*p* = 0.99). Moreover, only the disease activity correlated with lower scoring in the VAS of EQ-5D (*R* = 0.28, *p* = 0.03).  Figure 2 documents the correlation between different subgroups of disease severity of morphea and DLQI, with significantly higher DLQI scores in patients with severe activity of morphea. The presence of skin lesions on the upper limbs correlated in our patients with greater QoL impairment (higher DLQI score) (*p* < 0.001). We did not observe any significant relationship between the gender, subtype of morphea, disease onset, and duration and QoL impairment. Extracutaneous LS involvement, e.g., joint pain, also did not influence the QoL in our patients.

## 4. Discussion

For this study, a systematic search of the PubMed database was conducted with medical subject headings (MeSH terms) in various combinations: “morphea” or “localized scleroderma” and “quality of life”, “DLQI”, “dermatology life quality index” or “EQ-5D”. All achieved results were checked for relevance of the main topic, type of questionnaires of QoL, and type of scales used for severity of disease assessment. Ultimately, fifteen papers were taken for the final analysis. They were published between 2008 and 2019, and the number of patients ranged from 27 up to 581. Some of the studies used the Morphea in Adults and Children (MAC) cohort prepared by the University of Texas Southwestern Medical Center.

In the last 10 years, only 15 papers focusing on QoL among the patients with morphea were published. All of the studies have some limitations which are shortly summarized in Table 3 [11–25]. The major limitation of almost all of the previous studies is the relatively small groups of patients taken into the final analysis. It implicates rather poor diversity of morphea subtypes, with the highest prevalence of plaque-type morphea in the majority of studies. Only in 3 studies that the comparison of QoL between morphea patients and the control group, of either healthy individuals or patients with another dermatological disorders, is available [12, 17, 21]. In two of the studies, one performed on a pediatric population; no data regarding the disease severity assessment performed by a qualified physician was obtained [12, 15].

Except the abovementioned limitations, these studies give a further perspective on QoL among the patients with morphea, in which majority of them showed that morphea exerts a mild to moderate impact on patients' QoL. Modest differences in QoL were observed between the subtypes of morphea. As presented in the study by Das et al. and Bali et al., both disseminated morphea and linear subtype had higher, albeit not significantly, total scoring of the DLQI [18, 24].

The relatively small impact on QoL among the patients with morphea may indicate the need of a specific tool to assess specifically problems related to this dermatologic condition. Previously published papers that evaluated the influence of morphea on QoL gave undetermined answer, whether the subtype of the disease or its severity might have an impact on patients' QoL. In our study, we have conducted a new approach to classify morphea severity based on differentiation between mild, moderate, and severe diseases proposed by Teske and Jacobe [7]. Possibly, this division allowed us to show that severe morphea, in terms of activity but not damage, ultimately had an impact on patients' QoL. Similar results were obtained by Mertens et al. [22]. Previously, Das et al. observed that increased LoSSI and LoSDI scores correlated with a greater impact on QoL among the adult patients; however, he did not observe similar correlation between physician-based measures and CDLQI [18]. These results are similar to the one obtained in the study by Klimas et al. in an adult population, who additionally pointed out that physical disability, e.g., joint constrictions observed especially in the linear subtype of morphea, did have a negative impact on QoL [21]. Interestingly, significant differences between patients with pediatric-onset and adult-onset morphea were noticed in the study conducted by Condie et al. [20]. Based on three QoL assessment tools: SF-36, DLQI, and Skindex-29, the authors observed that QoL among the patients with adult-onset morphea was poorer comparing to that among patients with pediatric-onset disease [20].

In comparison with other dermatological conditions, pediatric patients with morphea have higher median DLQI scores than children with, e.g., systemic scleroderma [16]. However, as shown by Condie et al. patients with adult-onset morphea had SF-36 component summary scores similar to those seen in other dermatologic conditions, including psoriasis or atopic dermatitis [20].

Some of the studies focused also on additional symptoms of skin involvement in the course of morphea. Both Kroft et al. and Das et al. noted that the presence of fatigue, pain, and itch in the course of morphea significantly correlates with the QoL impairment assessed with the DLQI [13, 18].

In July of 2019, the results of the long-term, prospective, single-site MAC cohort study performed by Kunzler et al. was published [25]. This longitudinal analysis showed results from a 3-year follow-up, however, in the final assessment of either DLQI or CDLQI which took part only 38.7% of initial participants [25]. Authors received similar results to those published earlier that used MAC cohort for the analysis of QoL in patients with morphea; they observed only a mild effect of disease on patients' QoL. Although they underlined that functional impairment observed mostly among patients with the linear subtype of morphea might have important impact on QoL, that is not assessed by the DLQI or CDLQI.

The major limitations of our study are the relatively small group of patients with poor subtype diversity, which is a similar limitation as in previous studies. It is a result of a rarity of morphea and usual overall predominance of plaque-type disease [18].

The QoL in patients with morphea due to the lack of a good assessment tool dedicated specifically for morphea patients is hard to evaluate. As shown based on our results and in previous studies, the relatively small impact of QoL among the patients with morphea may indicate the need of a specific tool to assess this parameter specifically addressed to this dermatologic condition. This need might be finally fulfilled as new QoL measurement tools are in development. In 2019, Zigler et al. published a paper focusing on the development of a new health-related quality of life measure for individuals with pediatric morphea, which hopefully will contribute to a better therapeutic approach based on patient' expectations [26].

In conclusion, the evaluation of QoL in patients with morphea is still challenging due to the lack of good assessment tools dedicated specifically for morphea patients. In general, QoL in morphea patients is significantly correlated with the disease activity, but not with disease-induced skin damage. Further studies are needed to analyze QoL in morphea patients, especially regarding its rare clinical subtypes.

## Figures and Tables

**Table 1:** Demographic and clinical characteristics of the patient population.

	Morphea, *n* (%)
Gender	
Male	8 (12.3%)
Female	57 (87.7%)
Age	50.9 ± 20.5
Clinical subtype of morphea	
Plaque type	31 (47.7%)
Generalized type	15 (23.0%)
Linear type	3 (4.6%)
En coup de sabre	1 (1.5%)
Progressive facial hemiatrophy (Pary-Romberg syndrome)	2 (3.1%)
Atrophoderma of Pasini and Pierini (APP)	7 (10.8%)
Deep type	1 (1.5%)
Lichen sclerosus	5 (7.7%)
Autoimmune comorbidity	
Yes	14 (21.5%)
No	51 (78.5%)
Autoimmune diseases in the family	
Yes	14 (21.5%)
No	51 (78.5%)
Presence of antinuclear antibodies	
Yes	18 (27.7%)
No	24 (36.9%)
Not assessed	23 (35.4%)

**Table 2:** Level achieved in each domain of EQ-QoL-5D by patients with morphea.

	Mobility	Self-care	Usual activities	Pain/discomfort	Anxiety/depression
Level 1	90%	95%	82%	52%	53%
Level 2	7%	3%	15%	**35%**	**33%**
Level 3	3%	2%	3%	**10%**	**10%**
Level 4	0%	0%	0%	2%	3%
Level 5	0%	0%	0%	2%	0%

**Table 3:** Summary of previously published papers focused on QoL in morphea.

Author	Number of patients	Assessment of disease severity	QoL scales	Conclusions
Kroft et al., 2008 [11]	*n* = 74 (patients > 16 years), no control group	9-item skin status scale from ISDL VAS (itch, pain, and fatigue)	DLQI	(i) Based on the DLQI, morphea had a small impact on patient's QoL (mean DLQI was 4.2 ± 5.1 points). (ii) Higher fatigue, itch, and pain severity were significantly connected with a lower disease-related QoL, although both pain and itch were observed in less than 1/3 of patients and were of mild intensity. (iii) The major limitation is the lack of control group.

Orzechowski et al., 2009 [12]	*n* = 32 (age > 8 and <18 years) vs. healthy children (*n* = 33) and children with atopic dermatitis (*n* = 21)	Disease severity was not assessed	KINDL CDLQI	(i) General QoL of patients with juvenile morphea was not different from healthy controls. (ii) There were no differences in the median KINDL total or subscale scores between juvenile morphea and atopic dermatitis groups and juvenile morphea and healthy control groups. (iii) The major limitation is the lack of disease severity assessment.

Kroft et al., 2009 [13]	*n* = 74 (age > 16 years), no control group	9-item skin status scale from ISDL VAS (itch, pain, and fatigue)	10-item anxiety subscale and the 6-item negative/depressed mood subscale of the ISDL	(i) 38% of patients showed levels of anxiety or depressed mood comparable to those of psychiatric outpatients. (ii) A strong correlation between higher levels of psychological distress (depressed mood and anxiety) and a more severe skin disease was observed. (iii) The major limitations are the absence of a control group and the fact that the disease severity was assessed by patients and not by the qualified physician.

Arkachaisri et al., 2009 [14]	*n* = 15 (first phase of the study) *n* = 27 patients (third phase), no control group	LoSSI	CDLQI	(i) The median CDLQI was 3.0 (IQR 2.0–3.0), and the mean was 3.79 ± 2.61. (ii) CDLQI correlated poorly with the mLoSSI. (iii) The major limitation is the absence of a control group and relatively small group of patients.

Saxton-Daniels and Jacobe, 2010 [15]	*n* = 27, no control group	Data not presented in the paper	DLQI	(i) The mean DLQI was 3.5 (range 0-12), showing only a small impact of the disease on the QoL of the patients with morphea. (ii) Adults with pediatric-onset morphea had lower QoL than other patients, especially if they also demonstrated functional impairment (*p* = 0.05) and/or a large number of lesions. (iii) The major limitation of this study was the absence of a control group and relatively small sample group.

Baildam et al., 2011 [16]	*n* = 28 (aged between 4 and 16 years with diagnosis of either localized or systemic scleroderma), no control group	Modified PRES form	CHAQ (physical function and pain) CDLQI CQOL CHQ-PF50 (parent assessment and social function)	(i) Median CHAQ and VAS pain scores were higher among children with systemic scleroderma. (ii) The median CDLQI was 5 (range 0-10) for the total sample, reflecting only a small impact of the disease on patients' QoL in juvenile morphea and SSc. (iii) The patients with juvenile morphea had higher median DLQI scores than the SSc group (5 vs. 3, respectively), although the difference was not statistically significant. (iv) Only a moderate impairment of QoL and physical function was observed. (v) The major limitation is the absence of any control group.

Szramka-Pawlak et al., 2013 [17]	47 morphea patients and 47 healthy individuals	LoSSI	LOT-R	(i) The study was well designed and conducted. (ii) The current and past levels of QoL in morphea patients and healthy controls were similar. (iii) No correlation was observed between morphea severity and the QoL.

Das et al., 2014 [18]	*n* = 277 (202 adult patients and 75 children) based on the Morphea in Adults and Children (MAC) cohort, no control group	LoSCAT	DLQI CDLQI	(i) Mean DLQIs among adults were 6.58 ± 6.14 for generalized morphea, 6.0 ± 6.1 for linear, and 3.6 ± 4.7 for plaque-type morphea; however, no significant association between the morphea subtype and DLQI was observed. (ii) Mean CDLQIs among children were 4.7 ± 5.0 for generalized, 4.7 ± 5.1 for linear, and 0.5 ± 0.7 for plaque-type morphea. (iii) There was no association between the morphea subtype and CDLQI. (iv) Children with plaque-type morphea had a lower score in the CLDQI, but this group was not representative due to the small number of patients (*n* = 2). (v) On average, children had a higher impact on QoL compared with adults. (vi) The major limitation is the absence of a control group.

Szramka-Pawlak et al., 2014 [19]	*n* = 41, no control group	LoSSI	Skindex questionnaire LOT-R (optimism level) 29-item Mini-MAC	(i) There was no association between the subtype of morphea and QoL. (ii) The major limitation is the absence of a control group.

Condie et al., 2014 [20]	*n* = 302 (68 adults with pediatric-onset morphea and 234 patients with adult-onset morphea (based on MAC) cohort), no control group	LoSCAT	SF-36 DLQI Skindex-29	(i) SF-36 scores: pediatric-onset patients had higher SF-36 scores for physical functioning (53.2 ± 1.6 vs. 45.1 ± 1.3; *p* = 0.003), physical role (52.0 ± 1.7 vs. 45.2 ± 1.2; *p* = 0.007), vitality (51.4 ± 2.2 vs. 44.5 ± 1.1; *p* = 0.005), and physical component summary (51.7 ± 1.8 vs. 46.2 ± 1.3; *p* = 0.043). (ii) The mean DLQI score for pediatric-onset was 4.5 points and for adult-onset was 6.3 points. (iii) Patients with pediatric-onset disease had more favorable scores as measured by Skindex-29 (16.3 ± 3.5 vs. 34.6 ± 2.4; *p* = 0.0003). (iv) Patients with pediatric-onset disease had more favorable quality of life scores for all measures that reached statistical significance. (v) The major limitation is the absence of a control group.

Klimas et al., 2015 [21]	*n* = 73	mRSS LoSCAT	DLQI SF-36 SCQ VAS (itch, pain)	(i) DLQI data from the same cohort was presented in Das et al., 2014 [18] (see above). (ii) Morphea had a negative impact on QoL that was similar to disorders such as eczema and rheumatoid arthritis, especially in the domain of emotions and mental health. (iii) Lesional pain was associated with higher impairment in the SF-36 physical component summary and DLQI. (iv) The major limitation is the predominance of plaque-type morphea over other subtypes.

Mertens et al., 2017 [22]	*n* = 35 (only patients with eosinophilic fasciitis), no control group	mRSS LoSCAT PhysGA-A PhysGA-D	DLQI SF-36	(i) The median DLQI was 3 (range 0-18), showing only a mild impact of the disease on the QoL of the patients with eosinophilic fasciitis. (ii) The physical functioning domain of the SF-36 and DLQI scores moderately correlated with the PhysGA-D, mRSS, and LoSCAT scores. (iii) The major limitation is the absence of a control group.

Ardalan et al., 2017 [23]	*n* = 80 (aged between 4 and 16 years), no control group	LoSCAT	CDLQI (dichotomization of the CDLQI)	(i) The mean CLDQI score was not provided. (ii) The median CDLQI was 1 (range: 0-17). (iii) Each additional extracutaneous manifestation increased the likelihood of QoL impact by 37%. (iv) Each month after the initial visit yielded 5% lower odds of QoL impact. (v) Cutaneous activity and damage did not consistently reach statistical significance. (vi) The major limitation is the absence of a control group.

Bali et al., 2018 [24]	*n* = 101, no control group	LoSCAT	DLQI	(i) Median DLQI scores for generalized localized morphea was 4.7 ± 5.0, for plaque-type morphea 2.8 ± 4.0, and for deep morphea 8.0 ± 9.9 points. (ii) QoL scores indicated a mild to moderate negative impact of morphea on QoL. (iii) The major limitation is the absence of a control group.

Kunzler et al., 2019 [25]	*n* = 581 (based on MAC cohort) comparison of linear subtype with other subtypes of morphea, no control group	LoSCAT	DLQI CDLQI	(i) The median DLQI for linear morphea (3, IQR 1.5-7) was similar to that for the generalized subtype (4, IQR 1-9). (ii) The median linear CDLQI for linear morphea was 3 (IQR 1-6). (iii) There was no association between lesion location in cosmetically sensitive sites and life quality in the linear morphea group. (iv) Presence of functional impairment in adult patients showed a trend toward greater quality of life impact (median DLQI (IQR) with vs. without, 5 (3-8) vs. 3 (1-7); *p* = 0.05). (v) The major limitation is the absence of a control group.

ISDL: Impact of Chronic Skin Disease on Daily Life; VAS: Visual Analogue Scale; DLQI: Dermatology Life Quality Index; QoL: quality of life; KINDL: English language version of the German Revised Children's Quality of Life Survey; PRES: Pediatric Rheumatology European Society; CHAQ: the Childhood Health Assessment Questionnaire; CQOL: Child Health-related Quality of Life; CHQ-PF50: Child Health Questionnaire; SSc: systemic scleroderma; LoSSI: Localized Scleroderma Severity Index; SF-36: Short Form-36; LOT-R: Cantril's Ladder Life Orientation Test-Revised; LoSCAT: Localized Scleroderma Cutaneous Assessment Tool; MAC: the Morphea in Adults and Children; Mini-MAC: Mental Adjustment to Cancer Scale; mRSS: modified Rodnan Skin Score; SCQ: Self-Administered Comorbidity Questionnaire; PhysGA-A: the Physician Global Assessment of Disease Activity; PhysGA-D: Physician Global Assessment of Disease Damage.

## Data Availability

The datasets generated during and/or analyzed during the current study are available from the corresponding author on reasonable request (email: justyna.m.szczech@gmail.com).
